# The role of gamified learning strategies in student's motivation in high school and higher education: A systematic review

**DOI:** 10.1016/j.heliyon.2023.e19033

**Published:** 2023-08-09

**Authors:** Elias Ratinho, Cátia Martins

**Affiliations:** a Department of Psychology and Sciences of Education, University of Algarve, Faro, Portugal; b Psychology Research Centre [CIP] of University Autónoma de Lisboa / University of Algarve, Department of Psychology and Sciences of Education, University of Algarve, Faro, Portugal

**Keywords:** Games, Cooperative/collaborative learning, Improving classroom teaching, Mobile learning, Teaching/learning strategies 21st century abilities

## Abstract

Gamification, defined as the integration of videogame components to promote a gameful experience, is increasingly being implemented in education with the aim of enhancing students' engagement and motivation. Accordingly, since 2010 it has constituted an area of growing interest for researchers and teachers. Following PRISMA 2020's methodology, a systematic review (SR) was conducted in November 2022 seeking to explore the influence of gamification strategies on students' motivation to learn. Having identified 548 articles, 40 studies were chosen based on the selection criteria set and analyzed to reveal that game elements such as points, badges and rankings are widely used to motivate students. From a theoretical perspective, gamification studies focus on the dichotomy of intrinsic and extrinsic motivation. The results suggest a positive influence of gamification strategies on students' motivation, although in the long run, such motivation can decline. Furthermore, the influence of a novelty effect and extrinsic rewards on motivation is identified, which can lead to greater motivation in the short term, followed by a decrease with further exposure to gamification. Future studies should focus on the influence of students' individual traits (e.g., gaming experience, openness to competition and cooperation) on gamification strategies. Moreover, long-term exposure to gamification as well as the novelty effect should be explored.

## 1 Introduction

The history of the term ‘gamification’, which is closely associated with technology, dates back to the 2000s [[1], [2]], allegedly being coined by Nick Pelling in 2002 [3], although around the same time, Benthem (2002) also proposed that any logical task can be gamified [4], while not long later, in 2008, Bret Terrill referred the word gameification in his blog as a strategy to improve engagement [5]. Unfortunately for Terrill, his spelling of the word did not stick. Significant interest in gamification started to grow in 2010 [6] in areas such as business [e.g.,[7], [8], [9]], health [e.g., [10], [11], [12]], education [e.g., [4], [13], [14]] and crowdsourcing [e.g., 15].

Gamification is defined as the use of (video) game elements in contexts that are not game-related (e.g., enterprises, schools) [6]. Game design elements can be hard to identify, and no consensus exists as to a specific list of features [6,16,17,18]. Nevertheless, several approaches [e.g., [18], [19], [20]] can help designers understand the effects of game elements in learning environments [21].

### 1.1 Gamification

The most recent definitions of gamification identify the promotion of gameful experiences as the goal of gamification [e.g., [17], [22], [23]]; otherwise, a gamified design will not attain its purpose [5,17]. A gameful experience can be understood as a psychological state resulting from the interaction of a perceived achievable and non-trivial goal with a feeling of autonomy, leading to high levels of engagement [[4], [24]]. This conceptualization is compatible with the construct of flow, a psychological state introduced by Csikszentmihalyi (1997) [25] to describe high feelings of immersion in certain activities.

### 1.2 Gamification in education

One context that has seen an increase in gamification is education, where it is considered an important tool to foster motivation [e.g., [26], [27], [28], [29], [30], [31]]. The gamification strategies applied in education, also known as gamified learning experiences, aim to promote a gameful state in students, which can be facilitated through the introduction of game elements in a learning environment [e.g., [5], [13], [17], [32], [33], [34]]. It has emerged through games and their mechanics and has been gradually explored in the school system [[6], [35]]. When it is applied effectively, gamification fosters motivation and can thereby encourage students to become more involved in their school tasks [36].

### 1.3 Gamified learning strategies in education

In education, technological tools that might be considered examples of gamification are increasingly being incorporated in classrooms. However, other game-inspired practices and techniques are also popular in education, including serious games, game-based learning, 3D simulations and virtual reality (VR). Therefore, it is important to distinguish three concepts: (1) gamification, referring to tools that integrate game elements to provide gameful experiences without being games in themselves [6,37,17,23]; (2) game-based learning, which concerns the application of full-fledged educational games aimed at motivating students [[38], [39]]; and (3) serious games, whereby the real world and games are combined through simulations that aim to develop certain competences in a safe and relatively low-cost environment [[40], [41]].

In educational settings, gamified learning experiences are often applied to promote engagement and motivation in the learning process, since they make a student's progress clear [42,14]. To promote a gamified learning experience, gamified services are rich in game elements such as rankings, levels, points, rewards [16], badges [43], quests [36] and storytelling [14].

Numerous gamified learning digital services already exist. One of the most popular is Kahoot!, a quiz-based platform featuring game elements like points, rankings, competition and cooperation [44]. However, gamified learning experiences are all over the place and there are apps for a wide range of school subjects (e.g., Codeacademy [45], a gamification-based platform that helps students learn coding languages; Khan Academy [46], a digital application that includes a variety of school topics, focusing on STEM).

Therefore, students can benefit from gamified learning experiences. The literature in this field reports a positive influence of gamification in several aspects, one of which is motivation. Considering that students in high school and higher education may show low levels of motivation and high dropout rates, it is crucial to examine closely the effects of gamified learning experiences on their motivation [47,48].

### 1.4 Self-determination and self-efficacy in gamified learning experiences

With this review, according to our goals, we aim to understand student's motivational effects of gamification considering two specific approaches (i.e., Self-Determination theory; Deci & Ryan, 2000; Ryan & Deci, 2000; and Social Learning; Bandura, 1977, 1986).

Self-Determination Theory suggests that people are moved, especially, according to three basic psychological needs (i.e., autonomy, competence, and relatedness) and if their needs are fulfilled, then a person will be motivated toward something [49,50,[51], [52]]. In the educational field, considerable has been provided to the importance of autonomy and its support to the learning process [e.g., 54]. Gamification can have an important contribution: for example, if a gamified design promotes autonomy support through the possibility of choosing an avatar or a mission, if includes social aspects as chats or teamwork to facilitate relatedness and if give instant feedback for an achievement to foster competence, then the gamified experience will promote the basic psychological needs and students will be more positively motivated [e.g., [53], [54]]. To be noted that this approach and the research have demonstrated the importance of autonomy in the motivational process [e.g., [50], [54], [55]].

Another relevant approach in the learning context is the Social Learning Theory (Bandura, 1977, 1986) [56], in which self-efficacy is one main concept and refers to an individual belief of being capable to be successful in a certain task. Self-efficacy beliefs are influenced by several factors (i.e., mastery experiences, vicarious experiences, social persuasion, emotional and physiological states).

Therefore, we want to understand how self-efficacy is studied and measured in gamified learning studies. Overall, by incorporating game elements that promote mastery experiences, provide feedback, offer incremental challenges, facilitate social interaction, and encourage goal achievement, gamification can contribute to the development and enhancement of self-efficacy beliefs [49,57].

Another relevant aspect are the psychometric instruments used to assess the motivational constructs in the field of digital platforms and its impact on education and learning. Quite often researchers develop surveys or other resources to assess motivation and other aspects (e.g., quality, usability, satisfaction), according to their curiosity or research questions [e.g., [35], [40], [58], [59], [60], [61], [62]]. So, an identification of the main instruments used in research, based in the previous theoretical approaches, can reinforce their importance in this specific field.

### 1.5 Research objectives

Gamification is often regarded as an appealing strategy that improves motivation and engagement [6]. However, not all studies agree with these effects, for instance, some suggest a decrease in motivation after gamification exposure [63,64]. Therefore, more research is needed to understand the motivational learning effects of gamification [16,65].

Considering the above, in this review we aim to explore the motivational effects of gamified learning experiences in high school and higher education settings, in view of their needs and profiles as well as the potential risks involved (e.g,. college students frequently miss classes, while high school students can manifest high levels of anxiety and dropping out [48] In response, gamification may help increase college students' motivation [66], and attendance rates and mitigate high school students’ anxiety and propensity to drop out [[67], [68]].

Although some reviews on this topic already exist [[15], [69],^70^], motivational effects have yet to be fully explored in a psychological perspective, while some features (e.g., technology, apps) continue to evolve. Furthermore, the potential of specific components of the aforementioned theories (e.g., basic psychological needs such as autonomy; relatedness; competence; self-efficacy belief) have not been explored in much detail in gamification literature in educational contexts. Instead, reviews focus mainly on motivation via a quantitative framework, reporting whether students are more or less motivated following a gamification experience. However, it is important to understand if (and how) gamified learning strategies foster extrinsic or intrinsic motivation (i.e. their quality) and serve as inputs to levels of self-efficacy. Thus, it is important to track the psychometric instruments used to measure motivation and to understand gamification's effects on students' motivation, especially within self-determination (i.e. basic psychological needs) and social learning (i.e. self-efficacy) theories, as well as to explore gamification's effects on high school and college students' motivation [47].

An SR can help identify gamification's effects on students' motivation and aid teachers in introducing the most efficient gamified learning strategies in their practice. Consequently, the present study aims to explore the following:

(1) The instruments used to assess gamified learning strategies and features in higher and high school settings, considering the key concepts associated with motivation (i.e. self-determination, autonomy and self-efficacy);
(2) The gamified learning strategies and features most commonly used in high school and higher education settings;
(3) The effects of gamified learning strategies and features on high school and higher education students' motivation, considering the key concepts associated with motivation (i.e. self-determination, autonomy and self-efficacy)

## 2 Methodology

An SR is a widely used method for literature research, which comprises a rigorous structure of action to contribute with a beyond analysis [71]. It enables the confirmation or refutation of theories and helps researchers identify gaps that can inform future research [72]. Considering this method's advantages, we believe this SR will prove very useful for researchers and teachers looking to enhance their use of gamified strategies.

### 2.1 Data collecting

This SR research was conducted in November 2022 and searched for studies in Web of Science, Eric and PsycInfo, between the first of January of 2013 and the first of November of 2022. The SR followed PRISMA 2020 guideline [51] to assure a rigorous and thorough process.

The following keywords were used: Gamification OR gamified OR gameful experience (only title); Education OR Learning; Motivation OR self-efficacy OR self-determination OR Autonomy; High School OR Secondary School OR college OR higher education OR University. The words “gamified” and “gameful experience” are in line with the most recent definitions of gamification [37,17,23].

#### 2.1.1 Inclusion and Exclusion criteria

As inclusion criteria we designated the following: quantitative studies, English as main language, papers exploring gamification features, articles evaluating motivation variables (e.g., self-determination, self-efficacy or autonomy), samples of high school or higher education students and school or academic learning environments.

We did not include outcomes that met the following requirements: literature review, qualitative studies, theses, books; gamification studies in areas beyond learning contexts; studies that do not include motivational variables; samples composed by participants who are not high school or higher education students; studies not published in the English language and research that only considers educational games, serious games, game-based learning and/or virtual simulations.

#### 2.1.2 Studies selection

**Fig. 1:**
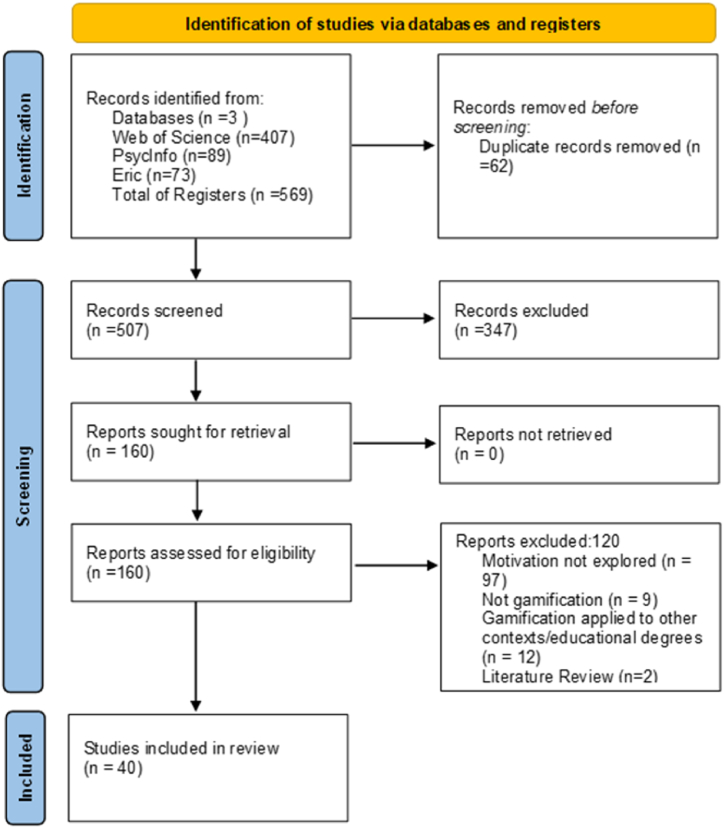
Flowchart of Systematic review.

As Fig. 1 shows, we found 569 articles, but 62 papers were duplicated, so 507 articles were analyzed. The Web of Science provided 407 articles, PsycInfo identified 89 articles and Eric 73 articles.

After a first analysis of titles and abstracts, we removed 347 papers (Table 1). The remaining 160 articles full texts were examined. After this first exploration, 120 studies were also removed because they did not meet the inclusion criteria, leaving 40 studies as analysis *corpus* in this systematic review (Fig. 1).

**Table 1:** Justification for excluded articles.

Exclusion criteria	*N* = 467
Literature reviews	58
Not gamification in learning contexts	155
Motivational aspects of gamification not explored	154
Higher education or high school samples not included	60
Not published in English language	17
Use other forms of educational games [e.g., game-based learning; serious-games; simulations]	23

## 3 Results and discussion

**Fig. 2:**
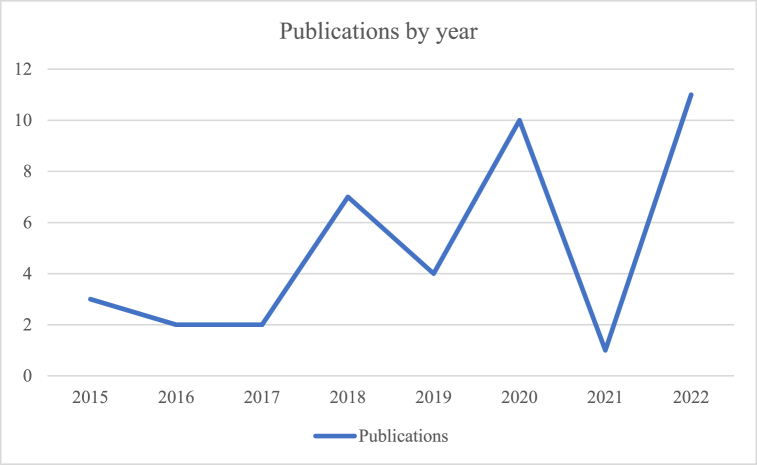
Publications by year.

A total of 40 studies were included in the SR, some of which were cross-sectional (14), but the majority were longitudinal studies (26), and 17 compared a control group to an experimental group. The majority were published in either the Spain or the United States of America (17), although a total of 17 countries were represented (Table 2), indicating widespread interest in gamification research. In terms of participants, the studies comprised between 22 and 683 students (*M* = 125.3; *SD* = 120.03), most of whom were in higher education; we only found four studies exploring gamification's effects on high school students' motivation. We also noted an increase in publications over time (Fig. 2), peaking in 2020 and 2022, revealing this topic's current relevance.

**Table 2:** Countries of the publications (*n* = 17).

Study from (Nation)	*N*
Spain	10
United States of America	7
Turkey	4
China	3
Germany	2
Saudi Arabia	2
Jamaica	1
Canada	1
Lithuania	1
Ireland	1
Singapore	1
Ecuador	1
Serbia	1
Taiwan	1
Belgium	1
Hong Kong	1
Finland	1
United Arab	1
**Total = 17 Countries**	**Total of Papers = 40**

### 3.1 RQ1: instruments used to assess gamified learning strategies and features in higher and high school settings

As Table 4 illustrates, Intrinsic Motivation Inventory (IMI) is one of the most widely used instruments to measure the influence of gamification in student's motivation [73,74,63,75,54,76,77,78,64]. This instrument which evaluates intrinsic motivation, specifically assesses Interest/Enjoyment, Perceived Competence, Pressure/Tension, Perceived Choice, and Value/Utility [[79], [80],^81^].

Both intrinsic and extrinsic motivation are among the most commonly explored motivational outcomes of gamification [83]. Therefore, gamification's effects from the perspective of basic psychological needs have been measured using the Academic Motivation Survey [44,84,85], Academic Self- Regulation Questionnaire [86,87], Student's Perceived Levels of Competence, Autonomy and Relatedness in a Gamified Flipped Class [88] and the Gamified Social Platform questionnaire [89].

Another set of instruments include the Self-efficacy and Learning Outcomes questionnaire [90] and the Patterns of Adaptive Learning Scales [91,92] from the Approach-avoidance Achievement Theory [93,94]. This theory focuses on the dichotomy of approach and avoidance. In brief, if an activity gives positive feelings, a person tends to keep on doing it (approach motivation). On the other hand, if the activity is undesirable and associated with negative emotions, a person will not do it (avoidance motivation). This theory states that students are motivated from either a performance or mastery perspective; for example, a student who is engaged in a task for the sake of learning is mastery-oriented, whereas a student who considers their grades most important is performance-oriented [95].

The Motivation Scale Course Interest Survey [96,90] and the Gamification Software Engineering Education Learning System (GSEELS) questionnaire [97] explore student's motivational levels through Keller's motivational ARCS model [98,[19], [99], [100]]. This model focuses on the appealing and states of an academic task considered motivating, where four conditions should be fulfilled: (1) attention; (2) relevance; (3) confidence and (4) satisfaction.

Other studies have explored student's motivational levels through focusing on gamification features, rather than measuring motivation through specific theories [44,101,89,57,102,97]. The Positive, Cognitive, Psychological and Instant Feedback Effects of Gamification scale [44] measure the cognitive and psychological effects of game elements in student's motivation, instant feedback, and sense of community. Another scale, adapted from the School Social Behavior Scale [SSBS; 115] [89], measures learning achievement, learning anxiety, motivation and autonomy within a gamification and flipped learning study. The Online Venture Challenge questionnaire [57] assesses gamification effects on student's experience, affective responses (e.g., enjoyment), engagement and self-efficacy. The Feedback Answers on Badges and Leaderboards questionnaire [102] examines student's classes attendance and academic motivation through badges and leaderboards. The Perceived Experience [97] explores gamification effects in student's motivation in a general perspective, along with features deemed enjoyable, engaging, fun, boring, challenging and relevant. Furthermore, the Physics Motivation Questionnaire [103] can be used to understand the effects of gamification on student's motivation in physics’ class.

To sum up, some authors have developed and adapted scales to examine the outcomes of gamification for motivation [104,44,89,57,102,105,97,106,88]; whereas others have used instruments inspired by motivational theories. For instance, self-determination theory [107,108,73,74,63,75,54,76,77,78,109,64,88], was used in used in 13 studies. In addition, the Approach-avoidance motivational theory [44,105] and Keller's motivational ARCS model [110,96,90,106,111] have also been used to evaluate the influence of gamification on students’ motivation to learn.

### 3.2 RQ2: gamified learning strategies and features most used in higher and high school settings

Educational gamified learning strategies (Table 3) are based on platforms and applications that use games elements, especially, points (75%), competition (65%), leaderboards/rankings (55%), and badges (52.50%). Other videogames characteristics explored are, for example, storytelling (22.50%) and levels (17.50%).

**Table 3:** Gamified elements in studies (*N* = 40).

Gamified Elements	Education Level		*f*	%
	High	Higher		
Avatar		35, 44, 106, 116	4	10.00
Badges/Status	98, 117	2, 3, 6, 11, 17, 22, 29, 34, 42, 44, 49, 54, 55, 62, 72, 82, 85, 92, 116	21	52.50
Coins		16, 22, 42, 55	4	10.00
Competition	117	3, 6, 11, 16, 17, 19, 20, 22, 30, 33, 34, 42, 44, 49, 50, 55, 67, 72, 91, 82, 85, 92, 99, 103, 108	26	65.00
Cooperation		3, 16, 17, 44, 49, 50, 55, 92, 97, 103	10	25.00
Instant feedback		44, 105, 106	3	7.50
Leaderboards/Rankings		6, 11, 17, 19, 20, 22, 30, 33, 34, 42, 44, 49, 50, 55, 67, 72, 91, 82, 85, 92, 97, 108	22	55.00
Levels	5, 98,	33, 54, 55, 82, 99	7	17.50
Points	5, 98, 117	2, 3, 6, 11, 17, 19, 20, 29, 30, 33, 34, 35, 42, 44, 49, 50, 54, 55, 67, 72, 91, 85, 92, 97, 103, 105, 108	30	75.00
Prizes		33	1	2.50
Roleplay		103, 105	2	5.00
Storytelling		35, 44, 50, 82, 101, 103, 105, 106, 116	9	22.50
Trading		16, 55	2	5.00

**Table 4:** Characterization of included articles [*N* = 40].

Nr.	Authors [Year]	Sample size	Education Level	Type of Study	Motivation Instruments		Gameful design		Gamification effects on Motivation
1	Aguiar et al., [2022]	69	Higher [Economic]	Longitudinal	Markov Model [Aguiar et al., 2022] based on Self-Determination Theory [SDT; Deci & Ryan, 1985, 2000; Ryan & Deci, 2000]		Points-based rewards and badges		Gamification fosters extrinsic motivation; however, extrinsic motivation can be internalized by students over time.
2	Aguilar, Holman, and Fishman [2018]	683	Higher [Undergraduate courses]	Longitudinal	Patterns of Adaptive Learning Scales [Midgley et al., 2000]		Points, badges, cooperation, and competition		Autonomy and competence increased in students that felt in control over the gamification grading system.
3	Al- Malki and Meccawy [2022]	60	High [Secondary school]	Longitudinal With control group	Instructional Materials Motivation Survey [Keller, 1987]		Points and levels		There is a positive effect of gamified learning activities in increasing students' motivation scores. The experimental group revealed higher levels when compared to the control group, where traditional methods were used.
4	Alabassi [2017]	47	Higher [Technology]	Cross-sectional	Positive, Cognitive, Psychological, and instant feedback effects of gamification [Alabassi, 2017]		Badges, points, leaderboards, and competition		Student's motivation, engagement and satisfaction were high after a gamified learning intervention. Instant feedback had an impact on learning motivation, participation, and academic achievement.
5	Asiksoy[2017]	61	Higher [Physics]	Longitudinal with control group	Physics Motivation Questionnaire [Glynn et al., 2009]		Points, badges, leaderboard, and competition		Student's motivation in the experimental group [gamification] was higher compared to the control group [flipped course].
6	Buckley and Doyle [2016]	156	Higher [Several graduation courses]	Longitudinal	Academic Motivation Survey [Vallerand et al., 1992]		Coins, trading, competition, and cooperation		Intrinsic motivation was found to be high within students using a gamification strategy. Extrinsic motivation was associated with a positive participation in the gamification tasks.
7	Cabot et al., [2020]	27	Higher [Master of Science]	Cross-sectional with control group	Gamified Social Platform [Engagement; motivation; involvement] [Cabot, 2020]		Badges, points, leaderboards, competition, and cooperation		Students felt the gamified platform was more motivating and an easy tool to learn, when compared to the control group. The social features of gamification [e.g., cooperation, participation in forums] were an important influence in the higher motivational levels.
8	Campillo-Ferrer et al., [2020]	101	Higher [Teaching Social Sciences]	Longitudinal	Motivation in Kahoot! [Ferrer et al., 2020]		Points, leaderboards, and competition		Student's motivational level increased after an exposure to Kahoot! competition system.
9	Cao et al., [2022]	156	Higher [Several graduation courses]	Cross-sectional	Reduced Instructional Materials Motivation Survey [RIMMS] [Loorbach et al., 2015] Goal Orientation Questionnaire [Xu et al., 2000]		Points, leaderboards, and competition		Students are more motivated when tasks are easier. Also, learning-oriented students reveal higher motivational levels than performance-oriented students.
10	Chen and Liang [2022]	187	Higher [Marketing]	Cross-sectional	Self-efficacy Speier and Frese [1997]		Not specified		There is a positive influence of gamification in student's self-efficacy to learn.
11	Chen and Zhao [2022]	272	Higher [STEM]	Cross-sectional	Perceived Locus of Causality [PLOC] [Ryan & Connell, 1989]		Competitions, study groups, coin/badge collections and rankings		Gamification fosters autonomous motivation which positively affects perceived usefulness and ease of use. Therefore, students feel comfortable and autonomy to use gamification apps.
12	Ding [2019]	70	Higher [Politician Education]	Cross-sectional with control group	Intrinsic Motivation Inventory [Ryan, 1982]		Badges and points		Students aware of gamification felt a higher sense of community, participation, and cognitive thinking.
13	Durrani et al., [2022]	105	Higher [Several graduation courses]	Cross-sectional with control group	Learning Motivation [Alavi, 1994; Leidner & Fuller, 1997]		Points, leaderboards, and competition		Students in the traditional group revealed higher motivational levels to learn than the gamified group.
14	Erümit and Yilmaz [2022]	52	Higher [Information and Communication Technology]	Longitudinal	Motivation and Engagement Scale-MES [Fredricks et al., 2005; Skinner et al., 2008]		Leaderboards, points, competition, levels, and prizes		Student's motivation was higher after the gamified intervention. Students described the gamified activities as an entertaining way of learning.
15	Facey-Shaw et al., [2020]	360	Higher [Programming]	Longitudinal with control group	Intrinsic Motivation Inventory [Ryan, 1982]		Badges, points, leaderboards, and competition		Student's intrinsic motivation from the gamified group decreased after the exposure to badges. Some students claimed to be frustrated and demotivated trying to unlock badges. However, in the control group, student's intrinsic motivation did also decrease. Authors suggested an influence of programming tasks complexity.
16	Ferriz-Valero et al., [2020]	127	Higher [Sports]	Longitudinal	Motivation in Physical Education Classes [CMEF] [Sánchez-Oliva et al., 2012]		Avatar, storytelling, and points		Student's extrinsic motivation increased [external regulation] after an exposure to ClassCraft. The experimental group [gamification] performed academically better than the control group.
17	González et al., [2020]	60	High [Secondary school]	Longitudinal with control group	School Social Behavior Scale [SSBS] adaptation [Yüksel, 2009]		Not specified		A comparison between gamified learning and flipped learning showed that both techniques could increas' student's motivation and autonomy and decrease learning anxiety.
18	Hanus and Fox [2015]	80	Higher [Communication]	Longitudinal with control group	Intrinsic Motivation Inventory [Ryan, Koestner, & Deci, 1991]		Leaderboards, points, badges, competition, and coins		Student's motivation decreased after an exposure to gamification, due to the competitive features of gamification [e.g., rankings] and to the long exposure to gamification.
19	Hazan et al., [2018]	91	Higher [Psychologic Statistics]	Longitudinal with control group	Intrinsic Motivation Inventory [Ryan, 1982]		Leaderboards, points, badges, avatars, storytelling, instant feedback, cooperation, and competition		Gamified condition led students to higher intrinsic motivation [perceived competence, interest/enjoyment, effort/importance] than the traditional learning condition. Motivational levels of students in the gamified condition were higher after the gamification strategy.
20	Isabelle [2020]	279	Higher [Arts and Commerce]	Longitudinal	Online Venture Challenge questionnaire [Isabelle, 2020]		Badges, points, leaderboards, competition, and cooperation		The gamified course increased student's motivation and self-efficacy.
21	Jaskari and Syrjälä [2022]	31	Higher [Marketing]	Cross-sectional	Game-playing Motivations in General [Yee, 2006; Yee et al., 2012; Kahn et al., 2015]		Leaderboards, points, storytelling, cooperation, and competition		This study explore student's motivation based on gaming personality: Social completionists; Highly motivated Completionists; Independent Completionists; Pure Completionists. Highly motivated completionists are always motivated, independent completionist motivation reduces in social elements of gamification, social completionists are highly motivated in social game elements and pure completionists are the less motivated in general.
22	Jones, Blanton and Williams [2022]	50	Higher [Kineosiology]	Longitudinal with control group	Intrinsic Motivation Inventory [Ryan, 1982]		Points, levels, and badges		In long-term exposure, both gamified and non-gamified groups lost interest in academic tasks. However, gamified group students revealed better autonomy and perceived competence than the non-gamified group students.
23	Jurgelaitis et al., [2018]	132	Higher [Informatics]	Longitudinal with control group	Intrinsic Motivation Inventory [Ryan, 1982]		Badges, points, leaderboards, competition, cooperation, levels, coins and trading		The gamified group was more intrinsically motivated than the control group. Interest/Enjoyment subscale was the most positively influenced by gamification.
24	Kyewski and Krämer [2018]	159	Higher [Several graduate courses]	Longitudinal	Academic Self-regulation Questionnaire [Müller, Hanfstingl, & Andreit, 2007]		Badges		Student's motivation level decreased in the gamified group but also in the control group [non-gamified].
25	Lopez-Martinez et al., [2022]	119	Higher [Physical Activity and Sport Sciences; Technicians in Teaching and Socio-sports Animation]	Cross-sectional With control group	Intrinsic Motivation Inventory [Ryan, 1982] [Escartí & Gutiérrez, 2001]		Leaderboards, points and competition		Gamified student's group intrinsic motivation increased compared to the traditional method; interest-enjoyment and effort-importance dimensions were high. Student's tension-pressure reduced after the gamified learning.
26	Mese and Dursun [2019]	63	Higher [Information Technologies in Education]	Longitudinal	Motivation Scale–Course Interest Survey [Keller, 1987]		Badges, points, leaderboards, and competition		Results of motivational levels [a–tention - relevance; confidence – satisfaction] were good and similar in both gamified group and control group. Qualitative analysis revealed an increase of motivation in the gamified group through the gamified experience; however, some students reported negative feelings with the experience due to lower places in the leaderboard and failures in the system.
27	Ortiz‐Rojas et al., [2019]	89	Higher [Programming]	Longitudinal with control group	Intrinsic Motivation Inventory [Ryan, 1982]		Leaderboards, points and competition		Student's intrinsic motivation [interest/enjoyment] did not increase in the gamified and control group. There was no difference between groups, neither a significant increase nor reduction of motivational levels.
28	Ozhan and Kocadere [2019]	40	Higher [Undergraduate courses]	Cross-sectional	Motivation Scale–Course Interest Survey [Keller, 1987]		Storytelling, levels, badges, leaderboards, competition, boss fights, and rewards		Authors explored the effects of flow and emotional engagement on motivation [ARCS model variables] through gamification. The results suggested an increase of motivation when students were more engaged and experienced flow.
29	Pinter et al., [2020]	282	Higher [Undergraduate]	Longitudinal with control group	Feedback answers [badges; leaderboards] [Pinter et al., 2020]		Leaderboards, points, badges, and competition		Students in the gamified group found badges and leaderboards to be motivating. The class attendance was higher in the experimental group [gamification] than in the control group. Leaderboard was motivating for most participants, but some students that were less competitive did not feel motivated.
30	Roy and Zaman [2018]	40	Higher [Master students]	Longitudinal	Academic Motivation Scale [Vallerand et al., 1992]		Points, badges, leaderboards, cooperation, and competition		Student's intrinsic motivation was negatively affected by gamification. In general, this study suggested that intrinsic motivation decreased over time in a gamified learning experience, and only the most extrinsic motivated [i.e., controlled motivation] remained stable over time.
31	Sailer and Sailer [2021]	214	Higher [Educational Social Sciences]	Cross-sectional	Short Scale Intrinsic Motivation [Wilde, Balz, Kovaleva, Urhahne, 2009] [82]		Points, team leaderboard, cooperation		Students revealed high levels of intrinsic motivation in this social-oriented gamified experience.
32	Sanchez et al., [2020]	60	High [Secondary Level]	Longitudinal	Not specified		Badges, levels, scores		Students exposed to the gamified experience were more motivated than the control group.
33	Santhanam et al., [2015]	182	Higher [Technology Mediated Training]	Cross-sectional	Self-Efficacy and Learning Outcomes [Santhanam et al., 2009]		Competition and levels		Student's self-efficacy beliefs, learning outcomes, engagement and enjoyment in the gamified activity were higher when they faced other students with the same level or lower level of comprehension/skills. In opposition, less competitive students, and students in competition against other students with higher abilities were less motivated.
34	Segura-Robles et al., [2020]	64	High [Secondary Level]	Longitudinal with control group	Basic Psychological Needs in Exercise Scale [Moreno et al., 2008], Sport Motivation Scale [Granero-Gallegos et al., 2014], and Sport Satisfaction Instrument [Baena-Extremera et al., 2012]		Storytelling and rewards		Intrinsic motivational levels increased in students from both groups [i.e., control with traditional learning and experimental group with gamified-flipped learning].
35	Stansbury and Earnest [2016]	93	Higher [Organizational Psychology]	Longitudinal with control group	Perceived experience [Stansbury & Earnest, 2016]		Points, roleplay, storytelling, competition, and cooperation		Students in the gamified group did not perform academically better than students in the control group [traditional learning]; however, students did engage and were more motivated and satisfied in the gamification condition. Individual preferences had an impact in gamification, some students were more motivated in competition.
36	Su [2015]	107	Higher [Software Engineering Education]	Longitudinal	Gamification Software Engineering Education Learning System [GSEELS; Su, 2015]		Points, roleplay, storytelling, and instant feedback		High degrees of gamification [deep gamification] improved student's motivation; however, gamification can increase student's cognitive load.
37	Tsay et al., [2018]	22	Higher [English Language Communication]	Cross-sectional	Intrinsic Motivation Inventory [Ryan, 1982]		Avatar, instant feedback, and storytelling		After one week of exposure to gamification, student's intrinsic motivation results were higher than average in enjoyment/interest and perceived choice [autonomy].
38	Valenzuela-Pascual et al., [2022]	60	Higher [physiotherapy]	Cross-sectional	Students' motivation and satisfaction [Escobar & Lobo, 2002]		Leaderboards, points, and competition		Most of the students were motivated after the gamification’ intervention and reported higher levels of motivation, when compared to the traditional educational approach.
39	Zabala-Vargas et al., [2021]	106	Higher [Engineering]	Longitudinal	Instructional Materials Motivation Survey [IMMS] [Loorbach, N., Peters, O., Karreman, J., & Steehouder, M., 2015]		Storytelling; avatars and status		Following the ARCS Model, students revealed high levels of motivation, however, in a long exposure to the gamified platform, student's motivational levels decreased [novelty effect].
40	Zainuddin [2018]	56	High [Science class]	Longitudinal with control group’Students' perceived levels of competence, autonomy, and relatedness in a gamified flipped class [Zainuddin, 2018]		Badges, points, leaderboards, and competition		Student's intrinsic motivation was higher in the experimental group [gamified] than in the control group. Competence, autonomy, and relatedness were fulfilled in the gamified experience, which led them to higher levels of intrinsic motivation and of participation in gamified activities; Students were motivated to unlock badges, gain points and to compete against each other.	

We also found that a variety of platforms are used in educational settings. “Gradecraft” [91], a learning management system that allows the integration of badges and points in academic tasks is one example. This software gives students the possibility to repeat tasks, affording them the autonomy and freedom to try again until they succeed. A similar platform is “EchoLu” [73], an online service with progress bars that rewards students with badges for successful tasks. A rapid response system named “Kahoot!” [44], is another application that turns the classroom in a competition show, in order to engage students in the learning process, through rewarding students who provide the most correct and fastest answers with a higher place on its leaderboard. “Who Wants To Be A Millionaire” and “Codeacademy”, which are also rapid response system platforms, have also been addressed by researchers. The use of quizzes platforms designed with game elements, like leaderboards and points, seems to be a widely explored method of gamified learning strategies [e.g., [78], [86], [101], [105]]. Some gamification experts [e.g., [64], [75], [106]] have developed gamified platforms that stimulate student's motivation through providing correct answering. The 2D video game-inspired app The Protegé [64], for example, promotes correct answering in order to progress in the narrative.

Although gamification’ platforms and applications are the preferred method, designers can also integrate game elements in the website Moodle, whether through the use of plug-ins allowing the inclusion of badges or points into this platform, to reward students for participating in a forum [101,74] or through lectures, articles and presentations [76,90]. For instance, in one study [109] points and badges were applied in Google + to explore gamification.

Overall, there is evidence that several types of gamified designs and techniques are used in educational settings (Table 3), which vary from simple approaches such as integrating badges or points [101,74,76,90], to more creative approaches [75,106,64] that include complex games features (e.g., roleplay, avatars, easter eggs, boss fights, quests).

### 3.3 RQ3: gamified learning strategies and features' effects in higher education and high school student's motivation

Gamification learning strategies are slowly being incorporated into classrooms and studies about gamification techniques' effects on student's motivation are growing (Table 4). Some research [57] has explored the novelty effect in gamification (defined as a pattern of high activity during the initiation of a gamified process, followed by a drop of activity after the novelty of the gamified activity disappeared) [64]. In one longitudinal study [57], the authors suggested that student's intrinsic motivation may decrease due to long exposure to gamified learning strategies. Such results caution of a negative association between gamification and students' academic performance, especially over a long period. Therefore, gamification can prove powerful in the short term, but once the novelty effect has disappeared, its extrinsic reward system may be unable to stimulate students' intrinsic motivation and even undermine their grade [74,63,86,109,111]. Another study [105], reported a decrease of student's motivation after a gamified experience; although in the control group, students' motivation also diminished, suggesting that this decline was not directly influenced by gamification, but rather by other factors, for example, individual differences (e.g., self-efficacy beliefs).

The opposite also has been reported, as some longitudinal studies have suggested a positive influence of gamified learning strategies on student's motivation. In one such study, points and badges were implemented over the course of three years [91]. In each year, the benefits of the gamified course were evident in every class, student's autonomy and perceived competence increased, and the participation in class task was positive. A similar study concluded that student's self-efficacy, engagement and motivation levels were high when compared to classes of previous years that did not experience a gamified learning strategy [57]. Another study [109] used a competitive gamification technique through the App “Who Wants To Be A Millionaire” and found that’ student's levels of immersion in the activities were high, leading to higher motivation and participation in classroom tasks.

On the other hand, most cross-sectional studies have reported high levels of student motivation [44,108,112,49,73,90,105], which may indicate that students are engaged and motivated to use gamified apps. In fact, when gamification is applied for a short period of time, gamified experience groups are usually more motivated than control group generally following a traditional learning method [44,108,90,105].

Gamification can also be competition-oriented or/and social-oriented. Some results [89,63,113,96,102,105] caution of a negative impact of gamified learning strategies in student's motivation, mainly due to social comparison and competition, showing the relevance of social-oriented strategies.

A decrease in student's motivation was reported in six studies [104,84,74,63,86,102,109], most of which used longitudinal designs and propose the influence of the novelty effect and individual differences. Nevertheless, most of the studies in this review reported an increase in motivational levels, showing that gamification regularly benefits student's motivation, at least in terms of how it is measured.

## 4 Discussion

The present work has explored a set of studies focused on gamification effects on student's motivation, regarding the key components of self-determination theory (i.e. autonomy) and social learning theory (i.e. self-efficacy), as well as the psychometric instruments used to measure motivation in order to highlight what is most effective and how. Several studies identified in this systematic review centred their analysis on gamification's influence on basic psychological needs, but it seems that self-efficacy has been relatively less explored, even though self-determination theory as well as the IMI have proved to be especially popular [81]. The influence of gamification in student's motivation has tended to be analyzed through qualitative rather than qualitative studies, focusing the basic psychological needs and types of motivation. The dichotomy extrinsic and intrinsic motivation as well as comparisons of student motivation between gamification and other learning strategies (e.g., traditional methods, flipped learning), represent the most commonly explored features of gamification studies. Most have used the IMI to report relatively high levels of enjoyment and autonomy when using gamification. This is evidence that gamification can lead students to a gameful experience, because features such as enjoyment, engagement and motivation are among the basic benefits it provides.

In terms of self-efficacy, this review did not find much work associating gamification with the effects of self-efficacy beliefs. However, some studies have found that the latter can be increased with gamified learning experience [57,105].

Although beyond our scope, besides self-determination theory and social learning theory (i.e. self-efficacy), we found that other theoretical approaches of motivation have been used to measure gamified learning experiences. For instance, approach-avoidance achievement theory [93,94] and Keller's motivational ARCS model [98,99] appeared in some of the articles included in this review [96,90,111]. This is evidence that, despite being relatively new, gamification is starting to be studied through several motivational theories, which we consider a very positive development.

However, there are also certain concerns regarding gamification, given that most rewards are boosters of extrinsic motivation, leading students to be more externally motivated and oriented [49,114,113,86]. Furthermore, gamification can foster competition, which risks increasing some students’ anxiety levels through social comparison; indeed, the use of rankings and achievements may give an advantage to more competitive students [86]. Competition certainly seems to be an influential variable in shaping decreases in student motivation, as some students appear to lose interest in gamification if they fail [49,113]. Students who find themselves low on a leaderboard or unable to unlock the same badges as their peers are at risk of increased anxiety and lower motivation [115]. Moreover, with regard to competition, some students can feel less motivated when they are competing [86]. Competition is one of the reasons for failure in gamified learning systems and, consequently, social comparison should be avoided [108].

Nevertheless, besides the negative feelings associated with extrinsic rewards and social comparisons, gamification can be successful in promoting motivation, especially under specific conditions. Gamification techniques must have a complex design and be challenging so that students can use these complex applications daily and play extremely advanced videogames, thereby achieving gameful states. However, for such a situation to occur, gamification designs must be appealing [90].

Another issue is the novelty effect of gamification. It seems that student's motivation decreases when they are exposed to gamified learning strategies for a long time [49]. By contrast, in short-term experiences with gamified learning, the results point to high levels of student motivation and satisfaction [91,101,108,75,86]. Declines in motivation occur especially after long periods of exposure to a gamified design, when the novelty effect disappears, and students lose interest in the gamification process [104,115,49,114,113]. There is, however, evidence that complex gamified designs can reduce the influence of the novelty effect, affording students higher levels of motivation, even with longer exposure to gamification [73,74,96,90]. In this review, the studies with the most complex gamification strategies [73,74,75,96,90,78] revealed increases in student motivation despite being longitudinal studies. This may be due to advanced gamified designs having specific conditions (e.g., alternate strategies, groups, and tasks) which readily facilitate gameful experiences and can thereby contribute to higher levels of motivation and engagement towards academic tasks.

According to this *corpus* of analysis, the most commonly explored game elements are points, rankings and badges. However, we also found gamification characteristics like storytelling and avatars, which shows that gamified learning experiences draw on a wide variety of resources and new ones are always being developed. These resources can help designers to effectively change and add game elements to their gamified techniques, in order to diminish the novelty effect and maintain students’ interest and achievement.

## 5 Conclusions

Despite this being the era of advanced technology, students still can exhibit low interest levels in (and for) their classrooms and task. In response, gamification may constitute a powerful motivational tool, combining video games, technology and academic content [[29], [116]]. Although gamified learning is still in its early days, gamification designers are trying to achieve a sense of autonomy, competence and relatedness in gamified apps to promote intrinsic motivation. Thus, the use of these apps in daily classroom activities or out-of-school activities may provide a necessary boost to the modernization of teaching strategies.

In an SR conducted in 2020, the authors concluded that gamification was beneficial for student's motivation and engagement over a short-term period of time [70]. Furthermore, Sailer and Homner (2020) meta-analysis revealed a significant effect of gamification on motivation to learn. These results are in line with the present SR, showing, that, in general, gamification increases students’ motivation to learn, even in a performance and external orientation.

Gamification is a strategy that foster extrinsic motivation and competition. Most game elements, as points and rankings, are competition-oriented although other game elements, such as badges, promote external rewards [117]. Long-term exposure can influence students' motivation. Furthermore, competition can decrease students' confidence and competence, mining one of the basic psychological needs and self-efficacy beliefs and diminishing its strategic potential [63,105]. Although these findings may cause some concerns, extrinsic motivation can be positive in some situations [81]: if a student is not engaged or cannot find purpose in some tasks or subjects, gamification can boost their actions [e.g., 88]. External rewards do not work for every student and can also lower already motivated students’ performance and motivation. Therefore, more studies are needed.

As limitations, we should note that our SR did not evaluate the quality of the articles included furthermore, we only explored high school and higher education students. Therefore, it is important that future SRs additionally assess more qualitative aspects of investigations as well as include other levels of education (e.g., middle school). We also noticed that there is still no consensus as to what gamification includes, as this learning strategy is often mistaken with serious games and game-based concepts. Consequently, future studies should explore the differences between these approaches and compare their motivational potential. The effects of gamified learning designs should also be addressed in the future, exploring and contextualizing the novelty effect. This gamification phenomenon has been identified as an important factor and a negative influence on students' motivation. Researchers must also explore the complex gamified designs' contributions to student motivation following a longer period of exposure as well as the role of students' personal traits in shaping gamification's success. The scope of this SR was small, rendering it necessary to explore other educational levels and compare across age groups.

With regard to future studies, we found that self-determination theory is the most frequently explored theory, so gamification research may benefit from exploring it further. Basic psychological needs can explain the effects of motivation on gamified learning strategies, namely when autonomy is supportive, but there are aspects associated with (for example) digital tools' social features that can have a greater impact on students' engagement and achievement. On the other hand, self-efficacy has rarely been explored in gamified learning experience contexts, so other constructs and approaches (e.g., attribution theory) could be addressed. Researchers are already studying this construct's relationship to digital tools in specific subjects (e.g., mathematics) so other subjects could also consider [118,119,70]. Other data analysis procedures (e.g., meta-analysis) could also be used in the future to complexify and add value.

In sum, gamification is a strategy that has the potential to modernize educational settings and promote engagement and motivation in students in the same ways that games do. Numerous game elements and characteristics can be used to engage students in learning. However, gamification can also affect students' motivation due to the use of extrinsic rewards, for example. Furthermore, students tend to react better to gamification when the process is new, whereas following a longer period of exposure, it can become less influential and even boring. For this reason, gamification needs to be explored further in education, but with caution.

## Ethics

This project was approved by the Scientific Commission of the Psychology and Sciences Education Department, University of Algarve, Reference No. E-UAlg/2020/20694.

## Funding

This work was funded by national funds through 10.13039/501100001871FCT - Fundação para a Ciência e a Tecnologia - as part of the project CIP - Ref^a^ UID/PSI/04345/2020.

## Author contribution statement

All authors listed have significantly contributed to the development and the writing of this article.

## Additional information

No additional information is available for this paper.

## Data availability statement

Data will be made available on request.

## Declaration of competing interest

The authors declare that they have no known competing financial interests or personal relationships that could have appeared to influence the work reported in this paper.
